# Supplementary material for: A Machine Learning Approach for the Discovery of Ligand-Specific Functional Mechanisms of GPCRs
Source: Molecules. 2019 Jun 2;24(11):2097. doi: 10.3390/molecules24112097 (PMC6600179; doi:10.3390/molecules24112097)
Supplement: Supplementary file 1 [file molecules-24-02097-s001.pdf]

A Machine Learning Approach for the Discovery of Ligand-specific Functional Mechanisms of GPCRs

Ambrose Plante [1], Derek M. Shore [1], Giulia Morra [1,2], George Khelashvili [1,3] and Harel Weinstein [1,3,*]

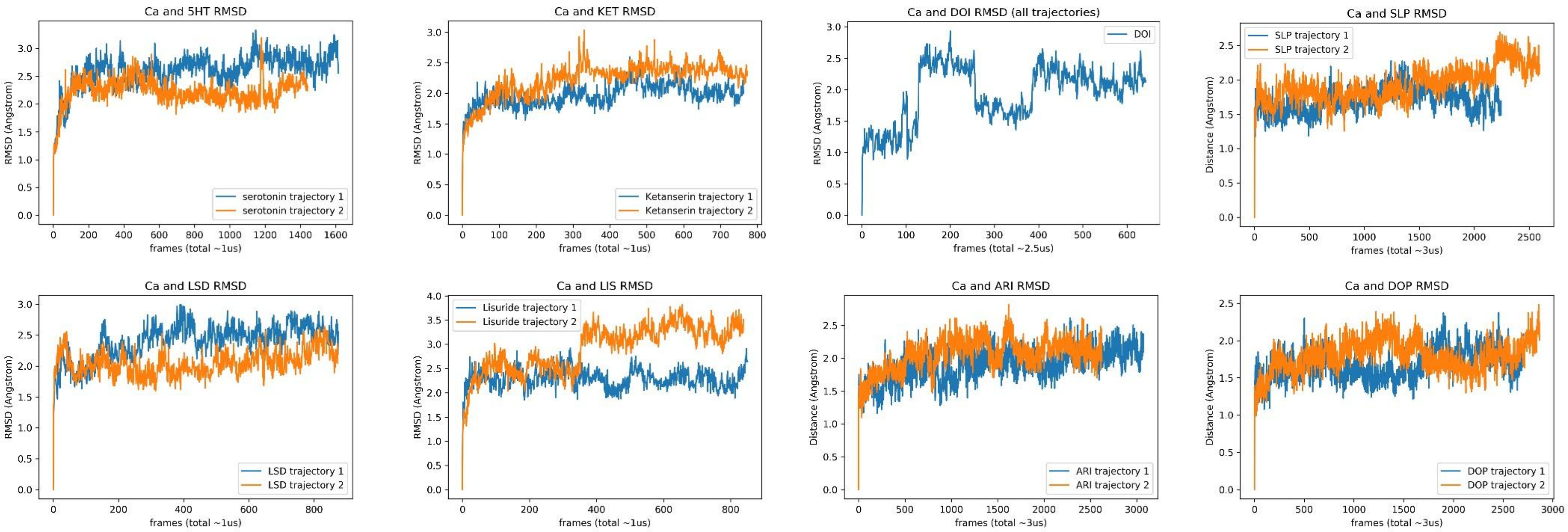

Figure S1. RMSD of the alpha carbon and ligand heavy atoms over the trajectories. The two trajectories of each ligand are colored in blue and orange, respectively, except for DOI for which 25 independently run trajectories are concatenated serially.
